# The Effects of Electronic Cigarette Emissions on Systemic Cotinine Levels, Weight and Postnatal Lung Growth in Neonatal Mice

**DOI:** 10.1371/journal.pone.0118344

**Published:** 2015-02-23

**Authors:** Sharon A. McGrath-Morrow, Madoka Hayashi, Angela Aherrera, Armando Lopez, Alla Malinina, Joseph M. Collaco, Enid Neptune, Jonathan D. Klein, Jonathan P. Winickoff, Patrick Breysse, Philip Lazarus, Gang Chen

**Affiliations:** 1 Eudowood Division of Pediatric Respiratory Sciences, Johns Hopkins School of Medicine, Baltimore, Maryland, United States of America; 2 Julius B. Richmond Center of Excellence, American Academy of Pediatrics, Elk Grove Village, Illinois, United States of America; 3 Division of Pulmonary and Critical Care Medicine, Department of Medicine, Johns Hopkins School of Medicine, Baltimore, Maryland, United States of America; 4 Division of General Pediatrics, Massachusetts General Hospital and Harvard Medical School, Boston, Massachusetts, United States of America; 5 Johns Hopkins Bloomberg School of Public Health, Baltimore, Maryland, United States of America; 6 Department of Pharmacology, Washington State University, Spokane, Washington, United States of America; University of Giessen Lung Center, GERMANY

## Abstract

**Background/Objective:**

Electronic cigarette (E-cigarettes) emissions present a potentially new hazard to neonates through inhalation, dermal and oral contact. Exposure to nicotine containing E-cigarettes may cause significant systemic absorption in neonates due to the potential for multi-route exposure. Systemic absorption of nicotine and constituents of E-cigarette emissions may adversely impact weight and lung development in the neonate. To address these questions we exposed neonatal mice to E-cigarette emissions and measured systemic cotinine levels and alveolar lung growth.

**Methods/Main Results:**

Neonatal mice were exposed to E-cigarettes for the first 10 days of life. E-cigarette cartridges contained either 1.8% nicotine in propylene glycol (PG) or PG vehicle alone. Daily weights, plasma and urine cotinine levels and lung growth using the alveolar mean linear intercept (MLI) method were measured at 10 days of life and compared to room air controls. Mice exposed to 1.8% nicotine/PG had a 13.3% decrease in total body weight compared to room air controls. Plasma cotinine levels were found to be elevated in neonatal mice exposed to 1.8% nicotine/PG E-cigarettes (mean 62.34± 3.3 ng/ml). After adjusting for sex and weight, the nicotine exposed mice were found to have modestly impaired lung growth by MLI compared to room air control mice (*p*<.054 trial 1; *p*<.006 trial 2). These studies indicate that exposure to E-cigarette emissions during the neonatal period can adversely impact weight gain. In addition exposure to nicotine containing E-cigarettes can cause detectable levels of systemic cotinine, diminished alveolar cell proliferation and a modest impairment in postnatal lung growth.

## Introduction

E-cigarettes also known as electronic nicotine delivery systems (ENDS) have become increasingly popular among current tobacco smokers and people who have never smoked.[1;2] However, a paucity of data exists on the *in vivo* distribution and systemic effects of nicotine solutions delivered by E-cigarette devices, or their potential for exposure in the nonuser. [3] Adverse consequences from E-cigarette exposure may be most pronounced in the very young. In addition to inhalation of expired constituents (nicotine, flavorings and carrier liquid), small children may also be exposed to E-cigarette toxins through breastmilk and contact with contaminated surfaces.

Secondhand and thirdhand exposure to E-cigarette emissions may be higher in young children who spend extended indoor time with a caregiver who is an E-cigarette user. Studies have been mixed regarding the likelihood of environmental contamination with the E-cigarette, since unlike conventional tobacco cigarettes E-cigarettes reportedly emit variable amounts of side-stream emissions. Nevertheless constituents exhaled from an E-cigarette user has been shown to contain indoor air nicotine levels similar to those of a vapor-generated smoking machine.[4] Indeed detectable levels of cotinine have been reported in subjects passively exposed to E-cigarettes with levels similar to those passively exposed to tobacco smoke. [5]

Airborne levels of nicotine and cotinine in homes of E-cigarette users have also been shown to be elevated above that of control homes indicating a secondhand exposure risk from E-cigarettes. [6] Due to higher respiratory rates, potential for greater dermal absorption and reduced nicotine clearance compared to adults, [7] neonates and infants are more likely to systemically absorb E-cigarette constituents including nicotine from the environment. Other factors that may increase the risk of toxicity in the neonate/infant exposed to E-cigarettes include close proximity to an E-cigarette user(s), consumption of breastmilk from an E-cigarette user and increased hand to mouth contact with surfaces contaminated with E-cigarette constituents. [8] The potential for multi-route absorption of nicotine and other components from E-cigarette exposure in the neonate and young child is especially concerning since rapid brain and lung growth is occurring during this developmental period.

The aims of this study were to determine if neonatal exposure to E-cigarette emissions would lead to impaired postnatal lung growth and systemic nicotine absorption. To address these aims we measured the impact of E-cigarette exposure on weight gain, postnatal alveolar growth and systemic nicotine metabolites in neonatal mice during the first 10 days of life. We found that neonatal mice exposed to nicotine containing E-cigarettes had detectable levels of systemic cotinine, decreased alveolar proliferation and impaired postnatal lung growth.

## Methods

Experiments were conducted in accordance with the standards established by the United States Animal Welfare Acts, set forth in NIH guidelines and the Policy and Procedures Manual of the Johns Hopkins University Animal Care and Use Committee. All experiments were approved by the Animal Care and Use Committee at Johns Hopkins University under protocol # MO12M255 approved 7/21/2014. Neonatal mice were euthanized by administration of isofluorane.

### Mice

Timed pregnant C57BL/6J mice were obtained from NCI (Bethesda, MD) and all experiments were performed in a C57BL/6 background. The animals were maintained under 12-h light/dark cycles in a clean environment.

### E-cigarette

Joyetech 510-T E-cigarettes were used for all experiments with 510-T tank cartridges, atomizer and auto switch battery. The E-cigarette solution used in the cartridges was obtained from Johnson Creek in 0% and 1.8% nicotine solution with no flavoring. Cartridges contained 400ul of solution.

### E-cigarette chamber and exposure

The E-cigarette puffs were actuated by a pump (Masterflex 7523–80 L/S Digital Peristaltic Pump Drive) and programmed to cycle every 15 seconds until the cartridge was empty (approximately 20 minutes). Each actuation was 6 seconds in duration and allowed E-cigarette emissions to move through the tubing and fill the chamber. The size of the chamber was 13.5 cm x 9 cm x 8.7cm. Exposure of pups began at 24 hours of life. Pups were kept with their mothers while exposed in the chamber and then placed back in their cages. Neonatal mice were either kept in room air (control mice) or exposed to 1.8% nicotine PG or 0% nicotine PG once a day for days 1 and 2 of life then twice a day from days 3 to 9 of life.

### Quantification of nicotine and cotinine in plasma and urine

Nicotine and cotinine levels in an aliquot of 10 μl sample were first spiked with 5 μl of D3 labeled nicotine and cotinine standard at 1ppm and then extracted by 30 μl of methanol with 0.1% formic acid by mixing and sit on ice for 10 minutes. The mixture was then centrifuged at 4°C for 10 minutes, and the supernatant was transferred to a HPLC sample vial and mix with 155 μl of 5 mM heptafluorobutyric acid (HFBA) for LCMS analysis.

The LCMS system used for nicotine and cotinine quantification consisted of an UPLC system (Waters) and a Xevo G2S QTof (Waters) mass spectrometer. An Aquity UPLC HSS T3 column (2.1x150mm, 1.8 mm) was used for the chromatographic separation. The column oven was set at 30°C. Solvent A was 1 mM HFBA and solvent B was 100% methanol. The LC gradient program was the following: 5% B from 0 to 2 minutes, linear gradient to 20% B from 2 to 3.5 min, 20% B from 3.5 to 6 minutes, linear gradient to 95% B from 6 to 7 min, 95% B from 7 to 9 minutes, and then equilibrate the column with initial conditions for 2 min with a flow rate of 0.4mL/min.

The mass spectrometer was operated in positive MS scan mode, with a source temperature at 120°C, desolvation temperature at 500°C, desolvation nitrogen gas flow rate at 1000L/hour, the cone gas flow rate at 50.0 L/hour, and the sample cone voltage at 30v. TargetLynx (Waters) was used for quantitative analysis. Quantification traces for nicotine, D3-nicotine, cotinine and D3-cotinine were m/z 163.12, 166.15, 177.1 and 180.12 respectively.

### Lung Inflation

Lungs were fully inflated with 1% low melt agarose at a constant pressure [9]. Lungs were fixed overnight in 4% paraformaldehyde. Lungs were paraffin-embedded, cut into five-micron sections and stained with hematoxylin and eosin (H&E).

### Mean Linear Intercept (MLI)

MLI measurements were performed on H&E-stained sections of lung and used as a marker for the distance between gas exchange surfaces.[10] Slides were coded, captured by an observer, and masked for identity. Ten to fifteen images per slide were acquired at 20X magnification by an observer blinded to the experimental groups. MLIs were assessed by digital morphometry with a macro operation using the NIS Elements software and recorded as arbitrary units. MLI was determined using the average distance between intersects of approximately 40 lines to the image.

### Immunohistochemistry (IHC)

IHC was performed on mouse lung tissue to examine KI67 expression. Lung sections were deparaffinized and rehydrate to water. Antigens were retrieved by heating the sections in BorgDecloaketRTU (Biocare medical, Concord, CA) for 10 minutes at 95C. After antigen retrieval, non-specific binding was blocked using Ultra V Block (UltraVision Detection System, Thermoscientific, Fremont, CA) for 10 minutes, then slides were incubated in humidity chamber with primary antibody from ABCAM (Cambridge, MA) at 1:4000 dilution for 1 hour at room temperature. As negative controls, adjacent slides were incubated in normal rabbit serum. After two buffer washes, slides were incubated for 10 minutes with biotinylated Goat Anti-polyvalent (UltraVision Detection System, Thermo Scientific), followed by a buffer wash and incubated for 10 minute in Streptavidin Peroxidase (UltraVision Detection System from Thermo scientific, Fremont, CA). After a buffer wash, staining of interest was developed with DAB chromagen (Dako, Carpinteria, CA) and counterstained with Fast Green (Sigma St. Louis, MO). Slides were then dehydrated and mounted with Cytoseal (Thermo Scientific, Fremont, CA). Quantification of immunohistochemistry staining was done as previously described.[11]

### Statistics

Differences in measured variables between treated and control groups were determined using Student’s t test (two-tailed, equal variance) or Mann Whitney U test. Statistical significance was accepted at p<0.05. Error bars represent standard deviation or standard error of the mean as indicated. Linear regression analysis with adjustment for sex and weight as appropriate was conducted using STATA IC 11.0 (College Station, TX) and co-efficient *p* values <0.05 were considered statistically significant.

## Results

### Total body weight was decreased in neonatal mice exposed to E-cigarette emissions

**Fig 1:**
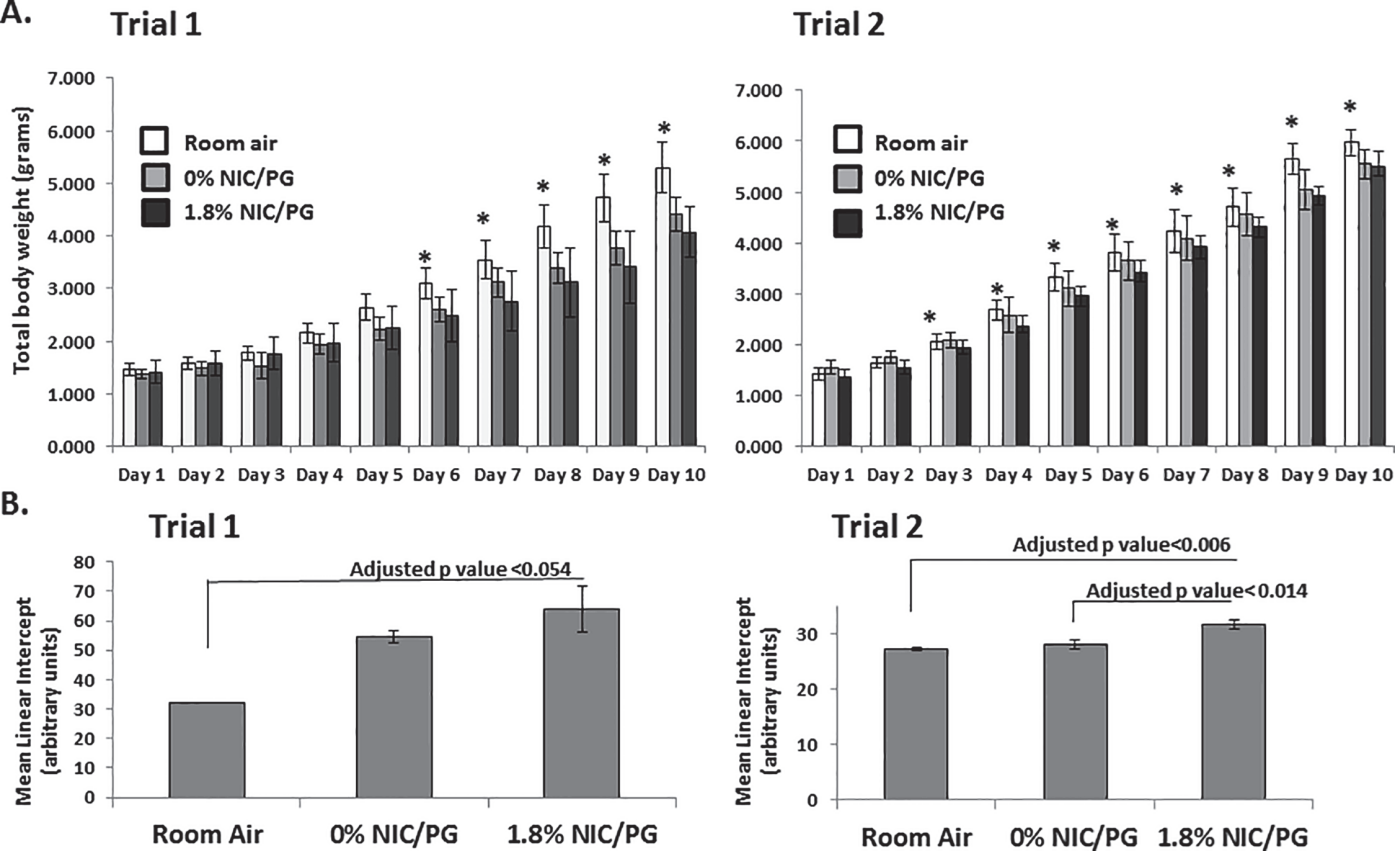
Effect of E-cigarette emissions on total body weight and mean linear intercept. **A.** Neonatal mice and their mothers were placed in a chamber and exposed to E-cigarette emissions starting at 24 hours of life. Age-matched control mice were kept in room air. Mice were exposed to either, 0% nicotine/PG or 1.8% nicotine/PG, once a day (400μl cartridge) for the first two days of life, then twice a day for an additional 7 days. Room air mice in trial one and trial two were significantly heavier at days of life 6 and 3 respectively compared to age-matched 1.8% nicotine/PG exposed mice (*p<0.05) and remained significantly heavier up through 10 days of life. (n = 5–13 per group, error bars reflect standard deviation). **B.** Neonatal mice exposed to 1.8% nicotine/PG had a larger MLI, after adjusting for sex and weight compared to mice exposed to room air (Trial 1: *p*<0.054 and Trial 2: *p*<0.006). In trial 2 neonatal mice exposed to 1.8% nicotine/PG had significantly larger MLI than 0% nicotine/PG exposed mice (*p*<0.014). (n = 5–8 per group, error bars reflect standard error of the mean).

Neonatal mice and their mothers were placed in the chamber only during the exposure period to E-cigarette emissions. E-cigarettes contained either 0% nicotine in propylene glycol (PG) or 1.8% nicotine in PG. Neonatal mice were weighed daily. In all groups, neonatal mice had a daily increase in mean weight up through 10 days of life, (**Fig. 1A**). At 10 days of life, mice exposed to 1.8% nicotine weighed significantly less than mice exposed to room air using linear regression and adjusted for sex, (Trial 1:*p* = 0.001; Trial 2: *p* = 0.003). The mice exposed to 1.8% nicotine/PG had a 13.3% decrease in total body weight compared to age-matched room air mice. In addition, mice that were exposed to 0% nicotine/PG also weighed significantly less than mice exposed to room air after similar regression analysis (Trial 1: *p* = 0.001, Trial 2: *p* = 0.008) with an 11.5% decrease in total body weight compared to age-matched room air mice. The decreased mean weight in the 0% nicotine/PG mice compared to room air controls suggest that nicotine alone did not entirely account for the lower weights.

### Mean linear intercept was increased in neonatal mice exposed to 1.8% nicotine

Alveolarization is not complete until 36 days of life in the mouse [12], we therefore measured mean linear intercepts (MLI) at 10 days of life to determine the impact of E-cigarette emissions on postnatal alveolar growth during a critical period of lung development. Mice exposed to 1.8% nicotine/PG had larger MLIs compared to mice exposed to room air after adjusting for sex and weight using linear regression analysis (Trial 1: *p*<0.054 and Trial 2: *p*<0.006), (**Fig. 1B**). In neonatal mice exposed to 1.8% nicotine/PG, MLI was not associated with cotinine levels in regression analyses adjusted for both weight and sex (*p* = 0.22). There was no difference in MLI between mice exposed to 0% nicotine/PG and mice exposed to room air after similar regression analysis (Trial 1: *p* = 0.27; Trial 2: *p* = 0.79). Mice exposed to 1.8% nicotine/PG had larger MLIs compared to 0% nicotine/PG exposed mice in trial 2 but not trial 1 when adjusting for sex and weight using linear regression analysis (Trial 1 *p*<0.34; Trial 2: *p*<0.014).

### Cell proliferation decreased in the airspaces of neonatal mice exposed to 1.8% nicotine

**Fig 2:**
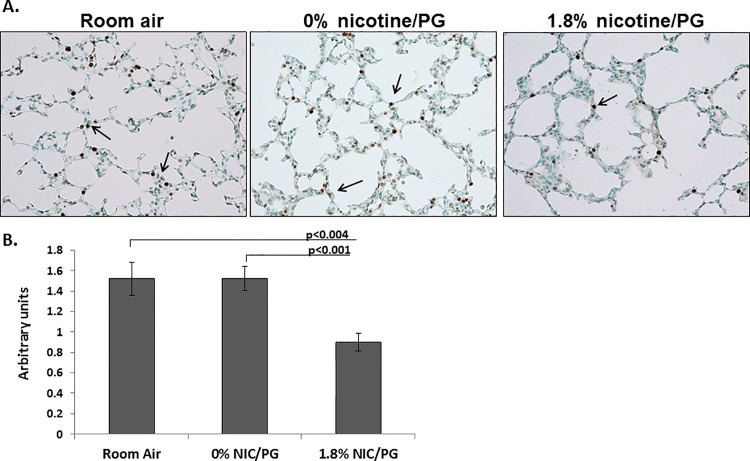
Decreased cell proliferation in airspaces of neonatal mice exposed to 1.8% nicotine/PG. **A.** Arrows point to KI67 staining in the airspaces of 10 day old neonatal mice. **B.** Quantification of KI67 staining showed significantly less KI67 staining in 10 day old neonatal mice chronically exposed to 1.8% nicotine/PG compared to room air and 0% nicotine/PG treated mice. (n = 8 per group, error bars reflect standard error of the mean).

To characterize the effect of E-cigarette emissions on cell proliferation in the alveoli of neonatal mice, Ki67 a marker of cell proliferation [13] was quantified by immunohistochemistry (**Fig. 2A and B**). We found significantly decreased KI67 staining in mice exposed to 1.8% nicotine/PG compared to room air and 0% nicotine/PG treated mice at 10 days of life, (room air versus 1.8% nicotine/PG, *p*<0.004, 0% nicotine/PG versus 1.8% nicotine/PG, *p*< 0.001) indicating that exposure to nicotine containing E-cigarette emissions can modestly impair cell proliferation in the alveoli of neonatal mice. However no quantifiable differences in caspase 3 (a marker of apoptosis) [14] or nitrotyrosine (a marker of oxidative stress) [15] were found between the different treatment groups.

### Plasma and urine cotinine levels in neonatal mice exposed to E-cigarette emissions

As a marker for chronic nicotine absorption, plasma and urine cotinine levels were measured in neonatal mice from trial one, (**Table 1**). Room air and 0% nicotine/ PG exposed mice had plasma cotinine levels less than 5 ng/ml and urine cotinine levels, less than 10 ng/ml at 10 days of life. In contrast neonatal mice exposed to 1.8% nicotine/PG had a mean plasma level of 62.3± 3.3 ng/ml and a mean urine cotinine level of 892.5± 234 ng/ml at 10 days of life. Using linear regression, adjusted for sex we found a significant association between total body weight and plasma cotinine level (*p*<0.033). In mice exposed to 1.8% nicotine/PG, each increase of one gram of weight was associated with a drop in plasma cotinine level by 13.45ng/ml. This finding suggests an association between lower body weight and higher plasma cotinine in neonatal mice exposed to nicotine containing E-cigarettes.

**Table 1:** Plasma and urine cotinine levels in 10 day old mice.

Plasma Cotinine				
Exposure	N	*Mean(ng/ml)*	*Range (ng/ml)*	*p value compared to 1.8%*
**1.80%**	6	62.3±3.3	49.3–70.3	-
**0%**	5	<5	-	<0.0001
**control**	7	<5	-	<0.0001
**Urine Cotinine**				
**Exposure**	**N**	***Mean(ng/ml)***	***Range (ng/ml)***	***p value compared to 1.8%***
**1.80%**	6	892.5±234	464.9–1903.9	-
**0%**	5	<10	-	<0.007
**control**	7	<10	-	<0.002

Neonatal mice exposed to 1.8% nicotine/PG containing E-cigarette emissions for nine consecutive days in trial one had significantly higher levels of plasma and urine cotinine respectively compared to 0% nicotine/PG exposed mice and room air control mice (± standard error of the mean).

## Discussion

In this study we demonstrated that neonatal mice exposed to 1.8% nicotine/PG containing E-cigarette emissions for one or two times a day had elevated plasma and urine cotinine levels and impaired alveolar growth at ten days of age. Neonatal mice exposed to 1.8% nicotine/PG had larger mean linear intercepts and decreased expression KI67 indicating modest but significant inhibition of alveolar growth in the nicotine exposed mice. Furthermore, neonatal weight correlated with plasma cotinine levels in nicotine exposed mice with higher plasma cotinine levels found in lower weight neonatal mice. Findings from this study indicate that exposure to nicotine containing E-cigarettes can cause systemic absorption of nicotine and modest impairment of lung growth during early postnatal life.

The high mean plasma and urine cotinine levels that were measured in the neonatal mice in our study, likely reflect nicotine absorption through multiple entry routes. Neonates and infants are more likely to absorb higher levels of nicotine and other E-cigarette contaminants through the skin. This is due in part to the larger surface area of skin relative to weight in the neonate and an immature epidermal barrier that can be especially prominent in preterm infants.[16] In addition, higher respiratory rates in neonates and infants can lead to greater deposition and absorption of nicotine through the respiratory tract. Also the high plasma and urine cotinine levels in the neonatal mice exposed to nicotine E-cigarette emissions may be due to the small chambers used to model an indoor environment in which an infant is in close proximity to an E-cigarette caregiver user. Furthermore, neonates and infants are more likely to ingest non-food items contaminated with nicotine.[7;8] Supporting this route of entry was a study that found that nicotine containing E-cigarettes can be a source for thirdhand exposure to nicotine. [17]

Toxicity from a substance may be heightened in the infant due to immature drug metabolism. [18] Dempsey and colleagues examined nicotine metabolism in newborn infants found to have cotinine present in their umbilical cord blood. They found that neonates had a nicotine half-life 3 to 4 times that of adults while cotinine half-life was similar to adults. [19] They also reported newborn blood and urine cotinine levels higher than100 ng/ml in several subjects during the first day of life indicating high nicotine exposure in some newborns. Spector and colleagues also recently reported elevated cotinine levels (range 0.5–247) from dried blood spots (DBS) of newborns of mothers who smoked. [20] Based on their previous study, in which the mean ratio of DBS cotinine to plasma cotinine was 1.49 [21], these levels indicate very high nicotine exposure in some neonates of mothers who reported a history of smoking. The higher levels of cotinine reported in these studies of newborns of smoking mother were in the range of what we found in neonatal mice exposed to nicotine containing E-cigarettes.

In our study, neonatal mice received their nutrition through breastmilk. Nicotine has been reported to be concentrated in the breastmilk of smokers.[22] Schulte-Hobein and colleagues found that breastfed infants of mothers who smoked > five cigarettes a day had urine cotinine levels ten-fold higher than bottle-fed infants of mothers who smoked.[23] Indeed they found that urinary cotinine levels of breastfed infants of smoking mothers could be in the range of adult smokers. Transmission of nicotine and or cotinine through breastmilk likely contributed to the elevated levels of systemic cotinine in our neonatal mice exposed to nicotine containing E-cigarettes.

There are potential limitations to our study. E-cigarettes reportedly emit little side-stream emissions, thus secondhand E-cigarette exposure is thought to occur primarily through mainstream emissions. Mainstream emissions however may pose significant bystander exposure. A model of particle delivery using an E-cigarette predicted that three quarters of generated particles containing variable amounts of nicotine, were exhaled [24] indicating that environmental contamination with nicotine and other E-cigarette constituents from an E-cigarette is plausible. In addition another study recently reported that air nicotine levels generated by a vapor producing smoking machine were similar to puffs from an E-cigarette user. [4] This indicates that secondhand nicotine exposure can occur with E-cigarettes and that a smoking machine can be used to model bystander E-cigarette exposure. Furthermore, E-cigarettes may also have side-stream emissions due to leakiness of the device. Supporting this assertion was a recent study that found formaldehyde levels similar to that of tobacco smoke when the voltage of the E-cigarette battery was increased from 3.2 to 4.8 volts.[25] Another challenge to our study was modeling the potential exposures of a breastfed neonate to an experienced E-cigarette user. To address these complex exposures we exposed neonates once or twice a day for approximately 40 minutes in a small chamber and used cotinine biomarkers to quantify nicotine absorption. Future studies will be needed to determine which routes of entry pose the greatest exposure risk in the neonate.

*In utero* and early postnatal life is a period of rapid organ growth and toxin exposure during this time period can potentially interfere with organ development.[26] In this study postnatal alveolar growth was modestly impaired in 10 day old mice following exposure to nicotine E-cigarette vapors, independent of total body weight. The nicotine exposed neonatal mice had an increase in MLI and a decrease in KI67 consistent with alveolar growth inhibition. Our findings support previous studies which reported impaired lung development in nicotine exposed mice.[27] Fetal and neonatal exposure to nicotine has also previously been shown to disrupt lung vascularization in a rodent model. [28] Maritz and Rayise reported that maternal nicotine exposure adversely affected lung development in Wistar rats, and at maturation histological lung changes resembled that of emphysema. [29] An analysis of studies examining the relationship between childhood respiratory health and pollution found associations between higher air pollution levels and adverse respiratory outcomes, in children with asthma. [30] These findings underscore the importance of examining the effects of E-cigarette exposure on vulnerable populations such as preterm infants and children with asthma who may be at increased risk for lung morbidities due to E-cigarette contaminants absorbed through the air, skin and oral ingestion.

In this study total body weight was decreased in neonatal mice exposed to E-cigarette vapors. Previous studies have reported that nicotine can cause weight loss or attenuate weight gain in people and in rodent models. [31;32] Nicotine exposure has also been demonstrated to have an adverse effect on prolactin release causing a decrease in breast milk volume. [33] Since neonatal mice were exclusively nursed during this study, we cannot rule out impaired lactation in the mother as a contributing factor to the lower daily weight gain in the neonatal mice exposed to nicotine containing E-cigarettes. The neonatal mice exposed to 0% nicotine/PG also had decreased weight compared to age-matched control mice suggesting exposure to the PG vapor alone may disrupt postnatal growth. At this time we do not have a biological explanation for the decreased weight in the mice exposed to 0% nicotine/PG. Since room air control mice were not placed in a chamber during an exposure we cannot rule out that some disruption of nursing may have occurred when pups were exposed to E-cigarette vapors.

In summary we found that exposure to E-cigarette emissions caused decreased weight gain in neonatal mice. In addition neonatal mice exposed to nicotine containing E-cigarettes had significant systemic nicotine absorption, diminished alveolar cell proliferation and detectable impairment in neonatal lung growth. These studies indicate that use of electronic nicotine delivery systems in close proximity to the very young may adversely affect weight and respiratory health.
